# Gift Game Metatheory: Social Interaction and Interpersonal Growth

**DOI:** 10.3389/fpsyg.2021.687617

**Published:** 2021-09-07

**Authors:** Gonzalo Alonso-Bastarreche, Alberto I. Vargas

**Affiliations:** ^1^School of Education and Psychology, University of Navarra, Pamplona, Spain; ^2^Mind-Brain Group, Institute for Culture and Society, University of Navarra, Pamplona, Spain; ^3^Hápax Action Sciences Institute, Ciudad de México, Mexico

**Keywords:** self-interest, utility, instrumental rationality, individualism, personalism, habits, moral psychology, game theory

## Abstract

This paper analyzes Game Theory (GT) from the point of view of moral psychology and makes explicit some of its assumptions regarding the human person as a moral agent, as well as the ends of human action, and reciprocity. Using a largely philosophical methodology, we will argue that GT assumes an instrumental form of rationality underpinned by a logic of self-interest, hence placing individuals, communities, and their social practices in service of external goods and their maximization. Because of this, GT is not adequate to describe the entirety of human social existence and interaction. Nevertheless, by revealing these assumptions, GT can be amplified with another form of rationality based on realist ethics and a personalist anthropology reinforced by the logic of gift. This rationality values the singularity of each person as a holistic unity, as the center of the social realm and as an end in herself called to growth and flourishing with others, nurturing the human community through giving and receiving. We will thus provide a wider philosophical framework for GT with a series of non-mathematical axioms of what can be called a Game Metatheory (GMt). These axioms refer to society as a complex system, not to particular interactions. GMt axioms are not a model of social games, but rather an axiomatic description of social life as a game, revealing its systematic character, complexity, and possible deterioration.

## Introduction

There is growing consensus around the idea that increasing complexity in organizations and society is one of the main challenges our era faces. Our understanding of human social action is in danger of disintegration both because of the many different disciplines that address it and the glut of analytic, partial approaches, rather than systemic and comprehensive ones, which frequently assess social action as individualistic. A disintegrated understanding reduces social action to result- or profit-centered interactions, thus seeing moral community, moral dispositions, and human flourishing as peripheral or irrelevant. Risks associated with this stance include the loss of long-term objectives and excessive organizational segmentation, the rise of unpredictable, perverse collateral effects due to partial views, and an increasing social anomie. In this context, the theoretical exploration of an integral approach to human social action seems necessary.

Surely one of the most successful tools for explaining human social action is Game Theory (henceforth GT). Despite GT’s remarkable development, its ability to overcome serious limitations, e.g., its limited consideration of moral and social dispositions like trust, altruistic cooperation, or love, and above all its ability to provide an explanation of moral community still remains unclear (Myerson, 1991, p. 370; Harsanyi, 1993; Binmore, 2005, 2007; Caillé, 2009; Carse, 2012; Gintis, 2014). However, the game structure itself still seems useful for describing human social existence and interaction (Deutsch, 1965; Boudon, 1981; Gintis, 2014; Polo, 2016a, pp. 105–132; Vargas, 2017b).

There are two opposite perspectives on the existence of agents’ moral dispositions and altruism within GT. While some authors consider moral dispositions unnecessary and altruism ultimately impossible for a rational agent (Binmore, 2005, 2009), others argue that human agency includes those dispositions, and that they are compatible with rational decision theory, even though classical GT itself still needs revision (Gintis, 2014).

This article aims to evidence some of the limits associated with GT assumptions from a philosophical point of view. We discuss limits, not defects. Wherever GT is applied to situations that conform to its assumptions, it is a wonderfully valid and predictive tool (Gintis, 2014, p. xii). However, following Gintis, we believe that social life goes beyond these assumptions. “Complexity [game] theory is needed because human society is a complex adaptive system with *emergent properties* that cannot now be, and perhaps never will be, fully explained starting with more basic units of analysis. The hypothetico-deductive methods of game theory and the rational actor model… must therefore be complemented… and develop insightful schemas that shed light where analytical models cannot penetrate” (Gintis, 2014, p. 196).

Game Theory appears to be especially adequate as a model for market interactions, but game theorists claim that a *game* refers to any social situation involving two or more individuals (Myerson, 1991; Binmore, 2005; Dixit et al., 2009). We argue, however, that this claim must be revised, especially Binmore’s (2005) attempt to ground social existence, social order and morality on GT. While social interaction can be described using a game structure, the philosophical foundations of GT require revision. In this debate, moral psychology has a lot to say. We assume a personalistic anthropological approach, considering the person herself as the center of the social realm and an end in herself called to flourishing with others. Thus, interpersonal growth becomes both the means and end of human action. We propose a series of non-mathematical axioms, a Game Metatheory (GMt), which refers not to particular interactions, but rather to society as a complex, open and free system.

This paper is divided into four sections: (1) A brief introduction to GT. (2) A critical analysis of GT’s assumptions. (3) Discussion of suggested alternative assumptions. (4) Discussion of GMt axioms.

## Game Theory Framework: Is There a Cooperative and Efficient Game?

To begin, we offer a brief introduction to the key concepts of GT^1^. An area of applied mathematics, GT can be defined as the analytical study of mathematical models of conflict and cooperation in situations in which two or more individuals with different preferences (or utilities) make decisions that will influence one another’s welfare. Centered on the notion of decision, its logical roots are found in Bayesian decision theory (Myerson, 1991; Binmore, 1993).

Game theorists describe agents’ preferences by means of a very abstract Bayesian version of the concept of *utility*. *Utility* refers simply to a specified ranking of agents’ preferences, or expected payoffs, in a specific situation (Ross, 2019). Those preferences are not identifiable with how much pleasure or how little pain a person may feel, or even with a sort of subjective psychological fulfillment or subjective welfare. Game theorists rely on concepts that are neither too psychologically normative nor human-restricted to describe agents’ preferences. In addition, given that even human agents often have more complex aims than simply earning money, utility cannot be simply identified with money. If and only if it is a scale, *utility* can be formally modeled in mathematical terms, and thus have a *utility function*. The simplest version of this mathematical device is the *ordinal utility function* (though there are more complex versions). Suppose that agent X prefers *a* to *b* and *b* to *c*. We then map these with a list of numbers, such that *a* = *3, b* = *2*, and *c* = *1*.

Given the *utility function*, GT stablishes a universal criterion for agents’ action: each player’s objective is to maximize the expected value of his own payoff (Myerson, 1991). This is called the *expected-utility Maximization Theorem*, the key concept in GT.

Rooted in this Bayesian foundation, GT considers that, “all situations in which at least one agent can only act to maximize her utility through anticipating (either consciously, or just implicitly in her behavior) the responses to her actions by one or more other agents is called a *game*. Agents involved in games are referred to as *players*” (Ross, 2019).

According to formal GT, if a situation does not fulfill these conditions, we can speak neither of a game nor of players. Some examples of non-game situations include monopolies, perfect competitions, or situations in which all agents lack defined utilities (e.g., because of a lack of knowledge), or cannot maximize them (e.g., because of mental illness, or even social marginalization).

Game Theory identifies utility maximization with *rationality*, that is, “a decision-maker is *rational* if she makes decisions consistently in pursuit of her own objectives” (Myerson, 1991). Philosophically, this is the riskiest, and therefore polemic, concept in GT. It comes from economic literature; thus, in strict terms, GT does not try to describe rationality in general, but only economic rationality, i.e., the rationality displayed by an economic agent in the market.

An economically rational player has at least (Ross, 2019) two alternatives in terms of her respective payoffs and can (1) assess payoffs, that is, rank them with respect to her preferences, (2) calculate paths to payoffs, (3) choose actions that achieve her most-preferred payoffs. This structure does not necessarily require deliberation; economic rationality can be embodied in behavioral dispositions built, for example, by natural selection. Therefore, GT involves behavior rather than reasoning.

Every player in a game faces various alternatives, called *sets of strategies*. Given that players are not alone, a strategy is a predetermined program of play that tells them what actions to take in response to every possible strategy that other players might use (Ross, 2019).

Strategies can be classified as superior/inferior, and dominant/dominated. Wherever one strategy gives more utility than another strategy, the former is superior. Wherever one action is superior to a player’s other actions *for every possible action by the opponent*, the former action strictly *dominates* the latter one. The dominant strategy for a player does not depend (or does so less than all her other strategies) on the opponent’s actions to maximize the player’s utility, while the dominated strategy depends more, sometimes even completely, on the opponent’s actions. The dominant strategy is not necessarily the superior one in any possible situation but is superior *regardless of what the opponent does*. There can be a superior strategy, but only insofar as it is coordinated with a specific opponent’s strategy. Domination thus refers to *independence* while coordination is aligned (perhaps too closely) with dependence.

In a game, different players have different preferences, which can be opposed or not. If players have strictly opposed preferences, they face what is called a conflictive, strictly competitive, or *zero-sum* game (there is a winner and a loser). But many real-life situations are not quite that simple: there are opposite preferences among individuals that still offer the possibility of deals and agreements that benefit everyone. And individual interests are not completely aligned either: players can reach an agreement and, when all opponents do what they are supposed to do, any one participant can get a better individual outcome by doing something different. If this is strictly the case in a game, sticking to the agreement is considered irrational. This is still considered a non-cooperative game [the Prisoner’s Dilemma (PD) is an example].

An important question arises: is a cooperative game possible? Myerson (1991, p. 370) clearly presents the difficulty involved. Cooperation means that, for two or more individuals *to act together with a common purpose*, they would have to set aside their separate utility functions and create something completely new–a collective utility function for determining their collective behavior. However, such a conceptual approach does not fit well within GT because of individual utility-maximization. Nash proposed studying cooperation by using the same basic concepts of non-cooperative GT, arguing that cooperative actions are the result of some bargaining process among “cooperating” players, and in this process each player is expected to behave according to her personal utility maximization. The pursuit of this is *Nash’s program*. So, cooperative games appear to be reducible to non-cooperative games. GT ultimately bases this reduction on the idea that conflict and cooperation “are two sides of the same coin, neither of which can be properly understood without taking account of the other” (Binmore, 2005, p. 58)^2^.

Game Theory’s main objective is to predict ‘rational’ players’ behavior once the utility function of every player is fixed and to provide the best possible solution. It seems obvious that the solution of a given game is different for every player because of their individual expected-utility maximization. Nonetheless, all players are co-implicated in the game, and thus the results of their strategies do not depend solely on themselves. Therefore, the solution must be a kind of *equilibrium* between players’ strategies. Though the concept of *equilibrium* is a very technical one, we aim to hold here to our analysis within moral psychology^3^.

The most important equilibrium concept is *Nash Equilibrium* (henceforth NE). A NE is a list of strategies, one for each player, such that no player gets a better payoff by changing her strategy, given the strategies of all opponents. It is important for us that NE does not provide the best payoff for every single player, or even jointly for all of them, but rather for everyone playing independently considering what the other player would do to maximize her utility given the other’s strategy. As an example, for the PD, the NE corresponds to both prisoners confessing and spending 2 years in prison, while the best for both is to deny, and spend only 1 year in prison. Therefore, in these kinds of games, NE is *inefficient*, while the *efficient* (or *Pareto-efficient*) solution finds no other outcome that would make all payers better off (Myerson, 1991, p. 97).

Game Theory has no difficulty in explaining why rational players should achieve NE, and even why some players do not (mainly because a lack of information or *non-rational*, utility-maximizing, behavior). The question remains as to how *rational* players can achieve *Pareto-efficiency* in a game. Of note, this involves a kind of *cooperation*, and individuals have no rational motivation to first choose cooperation against equilibrium. Explanations provided by GT of why and how players choose cooperation in one-shot games correspond to Nash’s reduction of cooperative games to non-cooperative processes of bargaining or the appearance of exogenous motivations like a penalty or leadership. However, if the game is repeated, the expectation of future major utility justifies cooperation (Dixit et al., 2009, pp. 397–414). The Gift-Exchange game, as a sequential version of PD (Charness et al., 2004), also provides a good example of this^4^.

Finally, while possibly trivial for mathematicians, philosophers will find it important that GT proposes quantitative models and hypothetical examples. Both are unrealistically simple in many respects, but this simplicity facilitates the comprehension of fundamental issues of interaction that are more difficult to see in the complexity of real life. This analytical method considers some details less important and ignores them to provide a simplified model. Nevertheless, “the price that has to be paid for an uncontroversial theory is that it cannot be used to model everything that we might like to model” (Binmore, 2009, p. 7).

## Game Theory’s Basic Moral-Psychological Assumptions

### Individualistic Logic of Self-Interest

Game Theory is frequently charged with relying on an individualist account of human beings because players are motivated by *self-interest*, formalized as a *utility-function*.

On one side, it must be made clear that the *utility-function* is formal, and thus does not refer to a kind of preference, but rather is applicable to any content, even altruistic, and players are not just individuals, but can also be corporations, etc. Therefore, all players are not necessarily *selfish* (Wendling and Viminitz, 1998; Binmore, 2005; Gintis, 2014; Ross, 2019). The entire issue of acquiring and fixing the *content of preferences happens before* a game interaction: “GT takes individuals’ preferences as given, regardless of the genealogy of those preferences” (Wendling and Viminitz, 1998, p. 37). That a player is *self-interested* does not refer to the specific content of her preferences: whatever the content of a player’s *self-interest*, she is self-interested if she does not overcome the difference between players’ preferences (i.e., cooperation *in* the game).

On the other side, we cannot reference a PD in this discussion because it does not embody the essence of human cooperation, and rather “represents a situation in which the dice are as loaded against the emergence of cooperation as they could possibly be” (Binmore, 2005, p. 63). If two players have aligned utilities in a PD, they would be playing what Binmore calls the Prisoners’ Delight game, which is actually a one-person, rather than a two-person, game (Binmore, 2005, p. 109). But then there is no cooperation.

GT individualism must be considered a *methodological individualism* (Gintis, 2014, p. xii), such that “it is not interests *in* the self, that take oneself as object, but interests *of* the self, held by oneself as subject” (Gauthier, quoted by Wendling and Viminitz, 1998, p. 38); they are self-interested but not self-regarding individuals (Gintis, 2014, p. 49)^5^. We can talk then of a morally formal, not material, individualism: players fix their *preferences* before game interaction, and the interaction between players consists only in achieving those preferences. Thus, once inside the game, a player is indifferent to the opponent’s preferences as such. This is not a trivial issue since “the most fundamental failure of game theory is its lack of a theory of when and how rational agents share mental constructs” (Gintis, 2014, p. xii).

Two questions then arise: (1) why are we to assume methodological individualism? And (2) how should we understand phenomena like love –or others that apparently go beyond *self-interest*, like altruism, or trust? Binmore succinctly responds to both questions in the following: “We are all separate individuals, each with our aims and purposes. Even when our capacity for love moves us to make sacrifices for others, we each do so in our own way and for our own reasons” (Binmore, 2005, p. 8).

The first question does not actually need to be answered by GT because methodological assumptions do not need to be justified in an analytical method; they just need to be useful tools for analysis. Any attempt at justification is usually a tautology: in a game, preferences are held by individuals, and players are individuals that have preferences in a game. Nonetheless, were someone to give an another justification of this assumption, the experience of opposed individual interests (and even conflict) is there to provide it (see Binmore, 2005, p. 101, 2007, p. 75). But experience also provides examples of the opposite, as Binmore also accepts, so we are just *choosing* one part, perhaps based on a preference. But then we cannot explain from within the model what we excluded from it at the very beginning.

The second question cannot be satisfactorily answered by ignoring that love includes behaviors toward the beloved, as well as care for her preferences, even intrinsic ones. If I take no interest in your interests, and if I do not include your preferences in mine, we can interact in society, but this interaction could not be called love. While it is useful for GT to exclude reference to *intrinsic preferences* and internal psychological causes of behavior (Wendling and Viminitz, 1998; Binmore, 2009), doing so excludes a dimension of human life.

From the perspective of *self-interest*, phenomena like altruism and trust are presumably reducible to individual utility in terms of a *reputation for honesty* (Binmore, 2005, pp. 8–9, 86–88): stingy players behave in an apparently altruistic way to keep up their reputation for generosity. Ross (2019) argues that this behavior can be maintained over time and remain effective. Nevertheless, a false reputation for generosity is fake altruism, so that explanation seems to be lacking. The extent to which a (false) reputation for generosity can be kept up in finitely repeatable games, not to mention throughout life in real society, is also unclear (Jackson and Kalai, 1999).

It is important to note that *methodological individualism*, as described above, is an anthropological issue (and not a properly moral-psychological, which would correspond to selfishness) since GT counts it as *the definition* of a human being (wherever players are human) (Vargas, 2017b). For GT, a human being is a player, a *self-interested* individual (though not necessarily selfish) interacting with other players. Is there anything anthropological beyond *self-interest*? For GT, nothing at all. Anthropologically, this definition is strictly individualistic because *self-interest* is chronologically prior to interaction, and this priority becomes an ontological priority: a human being is more properly an interested being than a being in relationship with other beings. However, this is assumed, rather than explicit, in GT, as seen above in the explanation of the terms ‘game’ and ‘players.’

The following limitations of GT are indeed aspects of this formal methodological individualism.

### Instrumental Rationality at the Service of Self-Interest

The GT concept of ‘rationality’ is *instrumental* (henceforth IR), that is, confined to the determination of means rather than ends: IR removes rationality from the sphere of ends [i.e., of questioning and discussing the (moral) quality of achievable ends], confining it to the sphere of means (i.e., of efficacy and efficiency; of finding the adequate means to achieve the ends previously proposed). While the appropriateness of means and ends for an agent is undoubtedly a matter of reason, it is hard to accept that finding this balance is reason’s only task, and thus has no role in determining the ends, evaluating them, or discussing them. This goes back to Hume’s famous claim in his *Treatise* that reason is, and ought only to be, the slave of passions, and that it is not contrary to reason to prefer the destruction of the world to scratching one’s finger. This confinement of rationality serves to avoid eternal philosophical discussions about the principles of rationality (Binmore, 2009), and provides a basis for a simplified model of rational decision. Nonetheless, it is worthless for understanding human action in a holistic sense.

To confine GT to IR, does not necessary mean previously confining rationality to IR. While the former is just a methodological assumption, the latter is a very difficult philosophical issue that touches on moral agency, metaethics, epistemology. Moreover, by confining rationality to its instrumental dimension, human rationality is nearly confined to individual efficient behavior (Binmore, 2009; Ross, 2019) or, even more contradictorily, to a decisional *attitude* toward one’s own efficient behavior (Harsanyi, 1993).

Binmore (2009, p. 6) explains that the ends-desiring passions, where not guided by reason, are at the same time rigid, inaccessible to external questioning, and unpredictable. The sphere of ends is properly the sphere of rational questioning and dialogue about the ends (why should I do this?). The sphere of means is not properly the sphere of dialogue, but of rapid application and efficiency. Thus, if there is no possible rational discussion about the quality or acceptability of an agent’s end, interpersonal dialogue becomes less possible, which leads to atomism. The lack of a theory about how we share mental constructs, which Gintis laments, overwhelmingly signifies a lack of interpersonal dialogue.

Moreover, accepting that only passions pose the ends of action implies that, “the pundits who claim to know the uniquely Good or Right way to do things are just blowhards; the reality is that we have only our likes and dislikes to guide us” (Binmore, 2009, p. 200). Even without asking if there is something inside us to guide us beyond our passions, it is possible to ask if passions really guide us. Spaemann lucidly argues, “The argument that everyone should do what they want… fails to take into account the fact that man is not pre-determined by his instincts but is a being who has to make a conscious effort to discover the principles which lead him to act the way he does… Unlike animals we cannot just ‘be’ if we are to be human; our lives are not simply automatic. We have, as people say, to ‘make a life for ourselves.’ We do have competing impulses and desires. The trouble with the maxim ‘do as you please’ is that it assumes that we know already what we want” (Spaemann, 1989, p. 9). The issue here is not finding someone to tell us what the unique good is, but that everyone needs to ask themselves and others, and look for an answer. Though the question may never be answered, we cannot make it disappear; if we try to banish it, we pay the price of external and internal conflict. It is true that moral discussions hardly reach agreement, but surely it is more difficult for two persons with different and merely passionate moral differences to come to a moral agreement when necessary.

### Mere Factual Interdependence Between Players

Aristotle explains the difference between *factual* and *moral interdependence* among individuals in society as follows: humans can come together for the sake of life merely, i.e., be *de facto* together, or be brought together by common interest, so far as each achieves a share of the good life, which is the chief aim of society (*Politics* III, 6, 1278 b 3 --1932, 201). Recovered in modern sociological thought by Durkheim, this distinction is nowadays very useful for addressing an important problem. *Moral interdependence* (henceforth MoIn) between individuals does not mean total moral agreement, which is in fact unachievable^6^, but a minimal moral community to care for the intrinsic preferences of others, and thus for their personal flourishing. Today, MoIn is also called *solidarity*, but this term is easily confused with mere ‘altruistic’ behavior.

As a matter of fact, *factual interdependence*, a product of the division of labor (Deutsch, 2020, p. 53), among other emergent effects (Boudon, 1981), impacts the interpersonal sense of community, whether positively, by enhancing *moral interdependence*, or negatively, by impoverishing it. The problem is that, in an economically globalized world, the following question arises: “How is the factual interdependence created by the division of work and increasing individualism related to the growth of personal consciousness and translated into a subjective feeling of community with other human beings?” (Stjernø, 2004, p. 320; see also Benedict XVI, 2009, p. 9). A ‘subjective feeling of community’ does not refer to a mere sympathetic reaction, but rather to having strongly entrenched subjective and reciprocal preferences for others’ well-being.

What does this have to do with GT? GT assumes players’ interdependence in a merely factual way: players interact based only on their own interest and according to their factual possibilities (usually considered economic power). Opponents’ *intrinsic preferences* are explicitly excluded (Binmore, 2009; Wendling and Viminitz, 1998), and that is, according to Gintis (2014, xii) and as previously mentioned, GT’s most fundamental failure.

Therefore, GT puts communities and social practices at the service of external goods and their maximization^7^. It is even possible to agree with Binmore’s argument that GT “makes a virtue of assuming *nothing whatever* about the psychological causes of our choice behavior… paying attention only to what people do” (2009, 8), and in so doing, “instead of disputing whose ethical or metaphysical system should triumph… we will be able to avoid getting entangled in numerous thorny paradoxes” (2009, 4). That is, the simplicity of behaviorism can be a useful tool, but it shuts out the possibility of explaining or, even less so, denying the existence of what is excluded, namely “internal” goods and their maximization. These internal goods correspond to players’ moral character and acquired moral dispositions (virtues), while interpersonal flourishing corresponds to the development of said dispositions. Do human rational agents have these dispositions, then? Do we really need them?

Gintis has extensively addressed the first question by recurring to many empirical studies testing behavior in games (2014, 48–78). He concludes that we have moral virtues as well as *other-regarding* preferences that influence our behavior in a game, even when they are costly for us, though we are ambivalent (i.e., not always other-regarding). Gintis’s development of GT is very thought-provoking in that it defends virtues and altruism, and considers individuals’ current state without abandoning the scope of rational decision theory. However, two possible limitations immediately stand out^8^. First, a strong distinction between character virtues and other-regarding behavior, considering virtuous behavior as merely desirable in itself (2014, 76). Secondly, from the point of view of moral psychology, what he calls *virtues* are not strictly such, that is, moral *habits* acquired through repetition of certain kind of actions that operate as individuals’ intrinsic principles. Rather, they are universal rules that operate as extrinsic moral principles, ‘‘a set of customary social norms that individuals often desire to follow simply because these norms are socially appropriate’’ (2014, 78)^9^. Therefore, “GT presupposes values as defined from the outside, as unmodifiable, and as independent of game results” (Deutsch, 1965). At best, some players are virtuous and other-regarding and will play accordingly, but game interaction does not enhance MoIn, which means no commitment to interpersonal flourishing. As noted, this would require a sharing of mental constructs, the lack of which Gintis (2014, xii) sees as GT’s main failing.

The second question, pertaining to whether we really need those moral dispositions, can be answered with a resounding “yes” because, widely shared, a minimum of moral dispositions is indispensable for interaction, and they are, as dispositions, not (only) prior to social interaction, but also develop in it. This appears to be clear in the phenomenon of *trust* and has been empirically proven: distrust generates distrustful people, and distrustful people mistrust themselves and become selfish (Weiss et al., 2018). In reverse, negative reciprocity arouses a spiral of distrust in repeated interactions (Harth and Regner, 2017). Interaction between distrustful people generates inefficient work routines (Bostedt and Brännlund, 2012).

Binmore (2005, pp. 81–82) suggests that trust can be replaced by fear of punishment. Apparently, in a game with rational players pursuing the only possible NE, “trustworthiness” refers to cooperation out of fear of being punished in a world made up of people unconcerned for their trustworthiness. Allegedly, in small societies, mutual knowledge allows for rapid punishment of deviations, and trustworthiness can be replaced, while in a modern city mutual knowledge is impossible, so *defection* is guaranteed. This claim reduces the issue to a problem of partial or complete information. However, that claim is still a theoretical assumption and has not been empirically proven. It is true that we are not always trustworthy, and punishment is necessary, and even more so as society grows larger. But is fear of punishment a good substitute for trust?

This seems theoretically impossible insofar as punishment is the mechanism for avoiding *defection* precisely when trust is impossible, such that it is not a valid substitute for trust whether in big or small organizations. A recent empirical study shows that punishment only guarantees trust and reciprocity in one-shot interactions (Wu et al., 2020). A meta-analysis across 18 societies clearly concluded that the effectiveness of punishment in promoting cooperation depends on the level of trust (Balliet and Van Lange, 2013). Moreover, because human physiology has separate neurological systems for responding to threats and facilitating social interactions, fear of punishment may inhibit some anti-social behaviors like cheating, but still not motivate prosocial behavior (Lenfesty and Morgan, 2019). In cases where players must have a personal commitment to fulfill their role, self-regarding actors who treat social norms as purely instrumental behave in a socially inefficient manner (Gintis, 2014, pp. 135–138, p. 205).

In addition, there is still one more major reason for which *factual interdependence* is insufficient: since we cannot live on auto-pilot, as if everyone automatically knew how to live and pursue the good life (Spaemann, 1989, p. 9), we need to share and discuss our *intrinsic preferences*. We in fact do so as part of social life, even though it does not fit within the GT model.

### Behavioral Rigidity

Allegedly, a game presupposes a fixed *utility function* so that it can be maximized. This structure involves a kind of rigidity: wherever the end of action (maximizing *this* utility) is fixed, the means (efficient strategy or behavior) can also be fixed. In addition, if preferences are not rational, and rather the result of passions, they can change, but cannot be improved upon or developed. This is because if reason is the slave of passions, they cannot be judged by reason, and thus cannot be better or worse.

Based on this scheme, either an agent behaves “rationally” or she fails to do so. Any move a player makes from fixed terms is considered irrational by most game theorists (Myerson, 1991; Harsanyi, 1993; Binmore, 2005, 2009; Dixit et al., 2009); some do not necessarily see it as irrational, but rather as the fruit of ignorance or misinformation (Gintis, 2014). Both views agree in essence. Any possible change during the game is a kind of *interference* in rationality because preferences and rational behavior are considered rigid.

In fact, human behavior is not rigid, but rather incredibly plastic: human beings do not behave without some subjective impact *from* their actions. “The principal victim of his action is he himself: he is a dynamic system endowed with an intrinsic *feedback*” (Polo, 2008, p. 86). This feedback is usually called *habit*, i.e., an acquired disposition of human behavior. Behavioral plasticity implies that players are modified *during* the game *because of* playing.

Our notion of habit is based on recent research from Bernácer and Murillo (2014, 2017). Some habits are only routinizations of patterns that can be acquired unconsciously– and at their worst extreme become addictions or compulsions. GT sees this behavior either as simple irrationality, or, more accurately, as a kind of rational behavior with the notions of state-dependent-preferences and time-inconsistency (Gintis, 2014). However, there are other kinds of habits, that enhance behavioral plasticity and cognitive control of actions (Bernácer and Murillo, 2014). They release consciousness from focus on immediate near-future goals and allow for all cognitive resources to focus instead on higher, distant-future goals. These habits were firstly proposed by Aristotle and can be called *habits-as-learning*. Since human cognitive or conscious control of action can be enriched (Bernácer and Murillo, 2014), *consciousness*, what GT calls the sphere of intrinsic preferences, or of mental constructs, is not a determined and immovable substance made of desiderating passions and its desired ends. Rather, it is a dynamic activity open to growth, as habits-as-learning *improve* consciousness (Bernácer and Murillo, 2017).

An example of these habits corresponds to the performance of a good artist, which opens up space for creativity, and more generally what positive psychology calls *optimal performance* or *flow* (Csikszentmihalyi, *Flow: The Psychology of Optimal Experience*, cited by Bernácer and Murillo, 2017). Another example is the acquisition of a healthy lifestyle (Bernácer et al., 2019). Moral virtues, e.g., the aforementioned disposition of *trust*, are also *habits-as-learning*.

Both kinds of habits can provoke a change in preferences during the game, but they cannot be considered in the same way. It is one thing to sacrifice the distant future for the near future, while the opposite is quite another. There is also much difference between diminishing self-control and improving it. Since *habits-as-learning* enhance behavioral plasticity and “increase the repertoire of actions and allow a better cognitive control of behavior” (Bernácer and Murillo, 2014), they can be considered a kind of development or growth.

### A Results-Centered Approach

For GT, every game has a final result of interaction that occurs when payoffs are revealed. The result of the game is external to it; during the game, there is no result, but when payoffs materialize, the game is over. Winning is only possible when the game is over. Once preferences are fixed, every player expects the conclusion. The game has a clear finalization for everyone. The result can be unsatisfactory for a player, but everyone sees it as more important than the game process. This different importance refers to a sort of logical priority, and thus can be called a *logic-of-result* (Vargas, 2017a).

### Conclusion: Game Theory Cannot Explain Social Existence

A critical analysis of GT does not imply whitewashing inconsistencies in individual behavior for a *new* kind of rationality (Binmore, 2007, p. 64).

Game Theory’s individualism does not correspond to the kind of selfishness that can destroy factual interdependence, but only to the *self-interestedness* that impedes cooperation in the game, which is incapable of enhancing MoIn. We do not believe that everything is either altruistic cooperation or selfishness, since competition among groups requires cooperation within these groups, and can be mutually beneficial among individuals (Gintis, 2014, p. 174).

In competitive interactions, GT modeling is very useful. However, empirical research on GT outside of perfect competitive market interactions shows that “game-theoretic predictions based on the *self-regarding* actor model generally fail. In such situations, the character virtues, as well as both altruistic cooperation (helping others at a cost to oneself) and altruistic punishment (hurting others at a cost to oneself) are often observed” (Gintis, 2014, p. 50). However, we will see later that the alternative to *self-interest* is not *altruism*, but *self-giving*). Therefore, starting from the premise that “how much people care about each other is an empirical question on which game theory is necessarily silent” (Binmore, 2007, p. 75) the only possible conclusion is that GT does not present a valid description of social existence.

For GT to explain social existence in general it must overcome the limitations described. Moral psychology, as an alternative and necessary discipline for understanding human teleological action and its goals can help by offering alternative assumptions.

## Alternative Assumptions From Moral Psychology

Overcoming the individualism of *self-interest*, GT’s very anthropological grounding, is not an easy task. As is well known, Alain Caillé and the MAUSS offer a strong proposal in this regard; they criticize the individualistic utilitarian theory of action and then offer a new paradigm for social sciences based on the logic of gift. Though their approach cannot be ignored here, some concerns arise that must be addressed^10^.

Against the absolutization of *interest*, Caillé (2009) offers a theory of action with four dimensions or polarities: self-regarding *interest*, *other-regarding interest*, *obligation*, and *freedom*. We agree that utilitarianism’s underlying monism is problematic (Caillé, 2009, p. 36), which can be overcome with a multidimensional theory of action. However, we think Caillé’s proposal has five problems.

First, his notion of *utilitarianism* is vague, it fails to distinguish between selfishness, methodological individualism, instrumental rationality, economism, and rationalism. He narrows the concept of *self-interest* to selfishness (2009, 21, 38–42). Second, these four *poles*, also called ‘motivations’ (in French ‘ressorts,’ ‘mobiles,’ 2009, 23, 33, 36, 60), can be considered internal psychological causes of action. As explained above, this is indifferent for GT’s concept of *self-interest*. Third, the poles seem philosophically vague. The concept of *freedom*, mere spontaneity and creativity, disregards the indispensable feature of *free will*. One could argue that *free will*, as the condition of possibility and core of all entire human action, preexists the four poles, but Caillé claims that the freedom-creativity pole is the core of action (2009, 61). Additionally, if the poles (especially freedom) make the acting human become a subject of social action (2009, 71), then the poles preexist humans before they become subjects. Thus, the poles appear simultaneously as cause and effect, and their ontological status remains unclear. Fourth, it is paradoxical that the constitution of the subject arises from the desire to become a subject (2009, 72), which certainly does not differ much from a kind of *self-interest*.

Finally, of note, Caillé’s proposal contains the basic structure of giving, receiving, and reciprocating. While reciprocation is necessary for explaining the exchange of goods from the logic of gift, it does not seem necessary for the basic ontological structure of gift.

To clarify the ontological status of the poles and to attain the ontological and anthropological structure of gift as superior to *self-interest*, a theory of the “not-yet subject” *acting human* is needed. Such a theory must be personalistic if it is to overcome individualism.

### An Interpersonal Logic of Gift

The core of personalistic anthropology surely corresponds to avoiding individualism by considering the person a being in relation with other persons (Melé and González Cantón, 2014, 178–203; Polo, 2016b; Murillo and Alonso-Bastarreche, 2018; Williams and Bengtsson, 2018). Having detected and explained GT’s individualistic assumptions, we assume personalism as the alternative anthropological assumption to GT’s individualism.

This assumption has two advantages. First, the notion of the individual is an abstraction because human beings are social beings; therefore, the individual is only acceptable as a methodological assumption, while the relational person represents real and extant human beings. Second, starting from the relational person offers an understanding of action that radically moves from a logic of *self-interest* to a logic of *gift* or, more precisely, of *self-giving* (Williams and Bengtsson, 2018).

Our personalistic approach is based on the anthropology of the Spanish philosopher Leonardo Polo (2008, 2014, 2016a; 2016b). This is first because the axioms we propose were first discussed by Polo, though not systematically (Polo, 2016a, pp. 105--132)^11^. Second, although it transcends the logic of *self-interest* and moves toward a logic of *self-giving*, Polo’s personalism does not neglect the former. One of this texts on this issue (2014) can be seen as a philosophical grounding for Godbout and Caillé’s (2000) thesis. Moreover, Polo’s personalistic anthropology seems capable of providing a fruitful starting point for the explanation of personal and interpersonal human agency (Akrivou et al., 2018) and for uncovering the remote anthropological foundations of economic activity beyond self-interest (Falgueras Salinas and Falgueras Sorauren, 2015). The person, according to Polo, not only has the subjective experience of executing action, but also produces it and receives it in the form of a habit^12^.

Polo describes the logic of self-giving as follows: “gifting is giving without loss, an activity that is superior to the equilibrium of loss and gain: gain without acquiring or acquiring through giving” (Polo, 2022). This means that the core of *self-giving* logic is not *what* the person gives or if she receives something in return, but that in such an act the person grows as a person; Godbout and Caillé (2000, p. 137) note that, “there is an immediate reciprocity of energy for the giver, she grows.” Whereas individualism hopes to find personal realization in *self-interest*, personalism seeks to make of the self a gift to another, and asserts the need for relational openness to others, even placing it as a condition for one’s own realization. The personalist approach is thus strongly committed to interpersonal flourishing.

According to Polo, the person beyond *self-interest* transcends IR, toward a form that can deal with *ends* and teleology; her *self-giving* capacity can be directed toward the natural world and toward other persons. When directed toward the world, *self-giving* means *contribution* in a strong sense, i.e., introducing novelties. When directed to other persons, *self-giving* involves reciprocal knowledge, attraction, communication, dialogue, and the possibility of cooperation (see Melé and González Cantón, 2014, p. 178). From this point of view, this logic is not opposed to *self-interested* interactions, but rather is compatible with them. The logic of gift is not mere altruistic behavior, which can ultimately be explained with the logic of *self-interest* (Faldetta, 2011; Polo, 2014)^13^. Thus considered, the capacity for *self-giving* is not a capacity for achieving certain goals, but rather a way of growing or flourishing unrestrictedly and developing interpersonal relationships (Faldetta, 2011; Polo, 2022).

From this perspective, prior to becoming a player in a game, a human being is a person, that is, a “being for others” or a “being with others,” which Godbout and Caillé (2000) similarly describe. Instead of assuming a game structure and then restricting anthropology, we begin the other way around. From this anthropological model, we then provide alternative moral-psychological assumptions.

### Ethical Rationality and Moral Interdependence

Following Weber (1978), within practical rationality, the complementary dimension to IR is usually called ‘ethical rationality’ (henceforth ER), though it could also be called ‘moral’ since it is tasked with guiding human activity. Does the expression, ‘*moral rationality*’ make sense? “In fact, morals is the most humane of all subjects… Since it directly concerns human nature, everything that can be known of the human mind and body in physiology, medicine, anthropology, and psychology is pertinent to moral inquiry” (Dewey, 1922, pp. 295–296).

Ethical rationality is complementary, rather than opposed to, IR. If we accept the possibility of an ER, it becomes possible to analyze and evaluate *ends* and, more importantly, rationally discuss them. For the individual, ER’s task is to find the best end for a particular action (‘‘What do I prefer? What I should prefer?’’); and for life in general (How should I live?’’). In social interactions, ER allows for moral discussion, which can certainly be understood as a kind of ‘negotiation’ between opposed moral preferences, but such an understanding eschews its essence. Indeed, it is closer to the essence of *dialogue* or *conversation*, i.e., several ‘players’ leaving aside opposite preferences and trying to share mental constructs (using Gintis’s expression)^14^. Every player is in principle open to changing her preference and to adhering to a new shared preference.

By accepting ER, it is possible, at least theoretically, to interact without having opposite preferences, that is, beyond individual preference. In this interaction, what really matters is *convoking*, *bringing together* players in a shared activity, and keeping the game alive as much as possible. This interaction requires a minimal MoIn.

As mentioned above, MoIn is a moral agreement by which one cares for the intrinsic preferences of other players, and thus for their personal flourishing, or in Gintis’s (2014, p. xii) words, a real sharing of mental constructs. Some characteristics of MoIn, as a kind of interdependence beyond a merely factual one, are described in what follows^15^.

MoIn is directed toward the interpersonal flourishing of players. This means caring for the progressive improvement and complexity of other players’ intrinsic preferences. MoIn is not compatible with mutual indifference, and therefore implies a certain dose of mutual knowledge. It also requires transparency (Melé, 2019, p. 128). MoIn implies caring for players’ human flourishing in interaction, as well as for their general well-being. It requires an openness to accepting new common preferences^16^. MoIn requires ER for each player to critically analyze her own personal convictions. One common example of MoIn is when a teacher discovers a talented student and tries to convince her to expand her horizons, proposing new and higher goals. MoIn is, in principle, *inclusive* in two senses. For one thing, it does not marginalize players based on economic power. For another, the more players the better. Of note, MoIn does not neglect scenarios of competitive interaction^17^.

### Behavioral Plasticity and a Process-Centered Approach

Given that human behavior is plastic and capable of enriching habits, *habits-as-learning* are important to GT because some psychological causes of behavior are not fixed (as explained in the context of *trust*), but rather are dispositions that are impoverished or enriched through behavior. GT has already indirectly faced these kinds of dispositions, i.e., what Gintis (2014, p. 205) calls *personal commitment* and *character virtues* correspond to them.

If players’ dispositions can be enriched, this enrichment must be considered for a complete understanding of human interactive behavior. As an example, the acquisition of a healthy lifestyle decreases effort-discounting in value-based decision making (Bernácer et al., 2019). From MoIn point of view, this enrichment is the most important goal of human behavior. Moreover, the process is more important than the result.

In common terms, ‘game’ refers to a kind of leisure activity, as Myerson (1991) laments. It is peculiar to this activity that its conclusion is always premature because nobody likes to conclude a game when she is enjoying it. This feature of leisure-based games, though not characteristic of the interactions analyzed by GT, can be extended to the entirety of life and society. In both cases, it makes no sense for players to expect a result *after finishing* the game (when there are no more players).

If the human person is capable of unrestricted growth or flourishing, a game’s successful end is always premature since the future remains open after every game and life continues (Polo, 2016a,b; Vargas, 2017b).

## Game Metatheory Axioms

These assumptions are an adequate basis for proposing a series of axioms that provide a more thorough understanding of human action in society. The axioms below, which have been extensively explored by Vargas (2017b), were inspired by Polo (2016a).

The term ‘axiom’ is frequently used as a synonym of ‘postulate,’ a statement stipulated to be true for the purpose of a chain of reasoning, that is, the starting point of a theory (Collins Dictionary, Encyclopedia Britannica). The term comes from the Greek ‘α*ξ*ίωμα’ meaning “to deem worthy,” which in turn comes from ‘α*ξ*ɩ*o*ς’ meaning “worthy.” The original meaning thus refers to a proposition taken as a starting point by virtue of its self-evidence, which in turn depends on its elevated epistemic and ontological value. Thus, axiomatization reflects both the ideal of scientific rigor and the intrinsic dignity of the subject of inquiry. Our use of the term is inspired in the former sense.

Since the *logic of gift* does not neglect the *logic of interest*, these axioms do not substitute those from GT. Indeed, it is important to neither neglect the GT model of human interaction, nor to consider it complete and sufficient. We aim to provide axioms for a wider framework and to propose a GMt. This GMt includes games explained by the logic of *self-interest*, though outlines the limitations of this logic. GMt axioms are also adequate for games that are irreducible to the logic of *self-interest*, which Vargas (2017b) calls Gift Games, since GT axioms do not work for this kind of game and for society as a whole^18^.

The following axioms do not refer to particular interactions, but rather to society as a complex system. GMt axioms are not a model of social games; they are an axiomatic description of social life as a game, revealing its systematic character, complexity, and possible deterioration. The universality of these axioms is as normative as ethics, and thus it is possible to violate them, but doing so damages social cohesion. After explaining MoIn, we assume that, “ethics is the only possible connective for social cohesion” (Esclanda and Sellés, 2016, p. 36).

We propose eight non-mathematical axioms that are, instead, descriptive (sociological) and normative (ethical) in nature.

### Axiom A: “We Play”

For this axiom, the game has already started, and it does not stop or finish. It holds for every player and for all. Nobody was asked to play the game—we just find ourselves alive and playing it. Since we are alive as social beings, our life is necessarily an interactive activity. This axiom can also be called the *activity* axiom.

As living, social persons, everyone is involved in a big interactive game called social life. In the phrase ‘we play,’ ‘we’ refers to everybody: *everybody plays the game*. This axiom has two intertwined meanings: a descriptive one that refers to factual interdependence, and a normative one that refers to MoIn. The descriptive meaning (‘social interaction simply exists’) is actually rich in implications, of which two stand out for our purposes here. First, just like *factual interdependence*, the game does not stop; players can play better and enhance MoIn, or deplete it, but the game does not stop, let alone finish. Second, even though everyone plays differently (i.e., some are better players), concluding that some people do not play is erroneous.

The normative meaning implies that nobody should be excluded from the game because exclusion negatively impacts both the excluded and the other players (Vargas, 2017b, p. 127). Spaemann (1989, p. 9) presents everyone’s major motivating factor for playing, namely living a human life means learning how to live since the good life is not a given.

Every player must be considered a *neighbor* for whose personal dignity and flourishing every other player cares; rooted in a personalistic account, we understand ‘*neighbors*’ as the people near to a person, with the moral significance of people who are lovable to her (Williams, 2005, pp. 302–320). Thus, opposition and competition must be restricted to particular scenarios such that people are not *exclusively* treated as competitors. If *the other* is considered a neighbor, and we do not want the game to end, then her playing the game is in my favor and, for her, it is also expedient that I play (Vargas, 2017b, p. 125).

If the game never finishes, then a player is called to face the entire game with hope because something completely new can happen in the game at any time.

Axiom A is chief among the remaining axioms, which are implicitly included in it, as we shall show below.

### Axiom B: “The Players (and the Moves) Are Different”

There are both many players and moves in the game, but distinguishing between them is not a numerical exercise; rather it relies on hierarchical and dynamic criteria. To understand this, we must first explain the concept of *type* (Polo, 2008). A *type* refers to a distinction that is neither personal, nor moral, but rather strictly social: psychosomatic differences of greater or lesser degrees, cultural differences, social classes, professional specializations, temperaments, the complexity of the decision-making processes, etc. *Type* differences refer neither to personal differences (a *type* can be shared by many), nor to moral differences (they do not imply moral-qualitative difference). Instead, *types* can be hierarchically and dynamically distinct.

Hierarchy refers to intensity, meaning that some types of players play more than others and some types of moves are better than others. In fact, in any given play, one or various types have more knowledge and power, and more capacity for action than others. Precisely because they are referred to as types, hierarchical distinction is dynamic; there is neither one unique superior person, nor one unique superior type: “If there is a plurality of persons of the same species, then there is a plurality of types. And this means to say that one person is superior to another in something, and the other is superior to the first in something else” (Polo, 2008, pp. 75–76).

While the best type of player has the highest capacity for action, the best type of move makes more and better future moves possible for everyone, which improves interconnection between future moves (the Pareto-efficient strategy).

The hierarchical distinction between players is not discriminatory, but just the opposite: everyone should respect and honor everyone else because any human being is superior to another in some respect or another. Hierarchical distinction of types does not imply social exclusion, but rather the opposite— continuation and improvement of the game. Distinction, when players are considered neighbors, makes it possible for players to care for one another (self-giving) and all players’ enrichment (that is, improvement of moral dispositions, ethical interdependence, and habits-as-learning). This synchronization is difficult to achieve. In the opposite case, a move would impoverish other players’ dispositions, damaging moral interdependence and leading to the game’s cessation.

Distinction is aligned with cooperation. Uniformity among players, or everyone thinking and behaving in the same way, is a sort of social malaise (Aristotle, *Politics* 1261 b 32). The game is only possible if players behave like neighbors, and this is only possible if they are different.

If players are neighbors, the entrance of every new player increases the possibilities for the rest, especially regarding future moves. On the contrary, reduction of the number of players, like their personal impoverishment, leads to tedium.

This axiom implies that, “the social problem is intrinsically ethical, it… has to do with considering the other as neighbor: ‘Thou shalt love thy neighbor as thyself.’ To love the other as oneself is simply to recognize him as neighbor: he is as much a person as I [am]; [his] type merits all my respect and appreciation. When we lose sight of this, the social problem becomes acute” (Polo, 2008, pp. 81–82). This axiom can be called the *neighborhood* axiom. The challenge of the social game is found in identifying strategies that diminish marginalization and enrich other players’ preferences and moral dispositions *in the hope* that other players do the same. Focusing on moves alone, leaving aside players and their intrinsic preferences, amounts to neglecting this axiom.

### Axiom C: “There Are Rules”

This axiom can be formulated as follows: players (and their respective moves) are irreplaceable, but they can be brought together by recurring to certain non-rigid rules. Every move belongs to a single player; thus, no player is dispensable. Even if a player belongs to a better type, she cannot substitute another one, and trying to do so can lead to social conflict. The available moves include *rules* that depend on the players, resources, and the level of synchronization that the players achieve.

The rules correspond to the information available to players: shared information among players, i.e., moral and cultural rules, that every player should respect. Since players are different, they require some synchronization; rules in a game exist to foster and increase synchronization or cooperation among players. Through cooperation, new and better strategies become available.

The rules must be as flexible as possible. Flexibility involves dynamism, the possibility of modification, and adaptivity. Flexibility does not in any way imply moral relativism; rather, the moral criterion for every situation is not *a priori* determined with a universal rule and instead requires agents to deliberate guided by the virtue of *prudence* or *practical wisdom*^19^.

No rule can be fixed because no player is fixed; every player’s behavior is plastic and constantly acquires enhancing (or impoverishing) habits. Moreover, players enter and abandon the game constantly. There is nothing like a *perfect and definitive rule*: players must be flexible and able to discover and redesign the rules again and again according to moral principles, and to leave aside rules that are no longer valid. Wherever a rule is fixed, the game becomes rigid, and players’ dispositions begin to decline. As mentioned above, the best rules for every move open up more possible moves for each future player.

Contemporary phenomena like globalization, information societies, social media, and artificial intelligence all impose some rules that players must know and follow.

Although they should be flexible, the game *must* include rules. Without them, and without information of any kind, players simply don’t know what to do and will abandon the game. Moreover, the rules must align with the game’s ends, be at the service of these ends, and put these ends in the service of players’ flourishing^20^. By doing so, the rules bring together (or *convoke*) players to a game that is ready to play. This axiom can be called the *convocation* axiom.

### Axiom D: “One Wins by Continuing to Play”

If the game does not finish, it is difficult to understand how to win it (see Carse, 2012). As a matter of fact, no one plays to lose; when a person knows that she is going to lose a particular game, they do not play if possible, or do not play fairly or thoroughly; “not even an inveterate gambler gambles to gamble” (Polo, 2008, p. 212). This is the descriptive meaning of this axiom, namely that *everyone plays to win*.

The normative meaning of this axiom refers to the problem of how to win, that is, what is there to gain from the game. Given that the game does not finish, that gain should, in turn, somehow emerge during the game. Indeed, it corresponds to nothing more than making the best moves and continuing to play better every time. The best moves make more and better future moves possible for everyone else, and enrich other players’ dispositions. In other words, winning means continuing the game by enhancing MoIn among players.

If this is what there is to gain from the game, everyone can win: “a social game is such a game that everyone plays, and everyone wins. Otherwise, it would be contradictory to be human and social at the same time” (Polo, 2016a, p. 123). Thus, this axiom can be adequately called the axiom of *joy*.

### Lateral Axioms

A lateral axiom makes explicit what is implicit in a central axiom. We mention four here, but there are more.

#### Axiom E: “One Does Not Play Alone”

Since society is a *factual form of interdependence*, play is not autonomous, and exclusion of one player negatively impacts the rest. Every player offers new alternative opportunities and strategies to the rest (though perhaps not too many). This axiom is a lateral axiom of central axiom A and can be called the *company* axiom.

#### Axiom F: “The Next Move Is the Best One”

The descriptive meaning of this axiom declares the primacy of the future over the present. In so doing, it reveals that the game does not finish and thus alerts us to the risk of expecting the game’s finalization. Its normative meaning can be easily confused with the utopian, unrealistic claim that, “the best is yet to come.” It actually points to the fact that, for a move to be best, it must open up more possible moves in the future. This axiom can be formulated in other ways, e.g., “success is always premature” (see Polo, 2016a, p. 111, 127) or the Augustinian claim that “if you sayest, I have enough, thou perishest” (*Sermon* 169, 15, 18). This axiom is a lateral axiom of central axiom B since its normative meaning specifies how the best move among a variety of moves is to be understood. It can be called the *unconformity* axiom.

#### Axiom G: “There Is a Board on Which the Game Is Played”

While social life certainly relies on non-material aspects, such as players’ dispositions, rules, and information, it also includes material resources, which constitute what can be called a *game board*. Is it reasonable to consider such a huge amount of material resources as a single board? According to the phenomenological description of practical agency, the term ‘*human world*’ refers to a structure of means, tools and material things shared by people:

The world, rather than being simply a complex unity of objects characterized by materiality, and extension, is in fact a network of meaning. More precisely the world we live in, and the world as we perceive it, is a world saturated by practical references of use. That the knife is lying there on the table means that I can reach and grasp it (Gallagher and Zahavi, 2008, p. 153).

The human being is not limited to having things, but rather communicates this having to the things he possesses. There are relations of what is possessed that are also intermediate, in accordance with which a human tool always refers to another. Up to a certain point, the things the human being makes possess one another mutually: they constitute a species of relational network. Because of this, a human world exists… which is a shared world because it consists of many related instruments. These interrelated instruments match the activities of a multitude of human persons, for whom this world is a shared world: a part of the common good. Human things are possessed in common, although for functional motives they are ascribed to certain individuals or to others; and this is property… There can be a virtuous use and a vicious use of private property (Polo, 2008, pp. 108–110).

Thus, if the game is social life, the board is the human world of instrumental means. If the game is to enrich others’ dispositions, then the world appears not as given but as *gift* for others. This is the basic frame to regulate the relationship between private property and common interest. It is not possible to take the board out of the game because it determines where and with what material resources we play.

Since the board is external to the players and brings them together, it is a lateral axiom of central axiom C. It can be called the *situation* axiom.

#### Axiom H: “By Playing, One Improves Everyone’s Game”

This axiom includes the plasticity of human behavior. Given that the game does not come to an end and that what is to be gained from the game consists in continued play, the game itself and players’ dispositions toward play can be improved upon. Dispositions toward play must be treated as habits because they are improved *while* players act. The game is thus an incredibly flexible structure, like social life itself. This axiom is a lateral axiom of central axiom D and can be called the *enrichment* axiom.

## Conclusion

Seeking to expand upon GT with an ethical and anthropological framework, we have suggested an axiomatic proposal related to a Gift Game Metatheory. We have done so to reduce the limitations associated with GT, namely the difficulty it has with building trust and cultivating love. From the perspective of moral psychology, we have taken on the challenge of rethinking social interaction from a personalist perspective (inspired especially by Leonardo Polo), which considers the person as a structural, open being related with others and capable of acquiring habits in a self-giving logic. Such a theory offers the guiding keys to society itself, pointing to its dignity and possible downsides. The convocation we have characterized here is essentially dialogical and interpersonal, and, in terms of a hopeful way of thinking, opens the way for a new theory related to leadership, government, and social action.

This proposal makes it easier to understand society as a complex reality, shining light on new alternatives for interaction, and on the discovery of a game related to interpersonal relations that is capable of unrestricted flourishing.

In the end, our proposal is a metatheory of human interactions based on the logic of gift and ethical rationality instead of on the instrumental rationality and methodological individualism that GT assumes. The advantages of this way of thinking include (1) the number of players is not restricted, (2) consideration of the human capacity for growth by acquiring habits, (3) integration of all of the possible games in the innermost part of the person and her relations with others, and (4) discovery and achievement of unrestricted interpersonal growth when what is to be gained from the game is properly understood. Keeping the game open and play running involves a deeper logic that transcends objectification of human reality and the need for a definitive result.

This proposal is still open and in need of further study, including further development of the axioms and exploration of its practical applications. It could also be enriched by critical comparison with Carse’s (2012) influential proposal, which also includes a theory of infinite games where what is to be gained from play is to keep the game alive.

## Data Availability Statement

The original contributions presented in the study are included in the article/supplementary material, further inquiries can be directed to the corresponding author/s.

## Author Contributions

GA-B wrote the sections “Game Theory Framework: Is There a Cooperative and Efficient Game?,” “Alternative Assumptions from Moral Psychology,” and “Game Theory’s Basic Moral-Psychological Assumptions.” AV wrote the sections “Introduction,” “Game Metatheory Axioms,” and final remarks. Both authors contributed to the article and approved the submitted version.

## Conflict of Interest

The authors declare that the research was conducted in the absence of any commercial or financial relationships that could be construed as a potential conflict of interest.

## Publisher’s Note

All claims expressed in this article are solely those of the authors and do not necessarily represent those of their affiliated organizations, or those of the publisher, the editors and the reviewers. Any product that may be evaluated in this article, or claim that may be made by its manufacturer, is not guaranteed or endorsed by the publisher.

## Footnotes

1 For a philosophical introduction, see Ross (2019). Dixit et al. (2009) contains a comprehensive mathematical and didactic exposition (see also Myerson, 1991). Binmore (2009) also presents a deep philosophical analysis.

2 Nonetheless, GT has standardized the terms *cooperative* and *non-cooperative* to refer to the way in which actions are implemented or enforced (collectively and individually, respectively), and not to the nature of the outcomes (Dixit et al., 2009).

3 For a technical explanation, see Myerson (1991); Binmore (2009), Dixit et al. (2009), and Ross (2019).

4 Thus, the question of “How can cooperation be promoted in a repeated PD?” (Askari et al., 2019) is already answered.

5 Askari et al.’s (2019) proposal of a hyper-rational player, does not overcome this framework. Such a player is just a rational other-regarding one (curiously, her other-regarding preferences can include the loss of others, such that she can be a hyper-selfish player).

6 If all members of a society were to think the same about common issues, it would be, in one sense, admirable, but still impractical and not at all a sign of concord [see Aristotle (*Politics* 1261 b 32 -1932, 77-)].

7 This is the consequence of the *logic of result* in Modern thought (Vargas, 2017a).

8 Gintis’s proposal requires more analysis, to which we attempt to partially contribute here.

9 For the difference between virtues and norms, see MacIntyre (1992) and Aristotle [*Nicomachean Ethics*, X, 9, 1179 b 25-35 (2014, 631)].

10 Despite this critical analysis, this contribution seeks to reinforce Caillé and the MAUSS’s interesting proposal.

11 Polo was inspired by his reading of Deutsch (1965) and Boudon (1981) when it came to his view of society as a game.

12 Let us mention a couple of clear philosophical advantages associated with Polo’s anthropology. He claims that, “the person is not limited to being, but she is co-being, co-existing, being-with” (Esclanda and Sellés, 2016, p. 31). Apart from naming personal relationality, this means that the theme of anthropology is distinct from that of classical metaphysics (*being*), yet compatible with it. The theory of knowledge necessary for dealing with this theme is quite complex (Polo, 2016b,a), and Polo’s approach can enrich personalist philosophy.

13 The logic of gift has been applied to economic activity, but a full discussion thereof goes beyond the scope of this article. A theoretical framework thereof can be found in Faldetta (2011) and Melé (2019).

14 Philosophy, mankind’s bi-millenarian endeavor, is nothing more than a huge historical conversation with no satisfactory solution (Bernstein, 1980); for those who see nothing beyond utility, it is the paradigm of irrationality (Binmore, 2005, p. x).

15 We provide some insights on this notion solely to provide a wider framework for GT. For a deeper approach to MoIn, see Stjernø (2004), Melé and González Cantón (2014, pp. 178–203), and Polo (2008).

16 This is very close to the concept of healthy organizations, see Di Fabio (2017).

17 The logic of gift does not exclude market logic (Faldetta, 2011; Melé, 2019).

18 The universality of GT axioms is extensively discussed. Deutsch (1965) discussed the universality of *transitivity*. Harsanyi (1993), with substantial experimental evidence that people often “*fail*” to conform to von Neumann and Morgenstern’s axioms, proposes a simplified and generalized version of them.

19 See Aristotle (*Nicomachean Ethics*, VI, 1140 b 1 ff.).

20 In an organizational context, implementation of a similar model has proven to be effective (Di Fabio, 2017).
